# Artificial neural network and machine learning predictive model for assessing physicochemical properties of garlic slices (*Allium sativum L.*) during microwave-assisted convective drying process

**DOI:** 10.1016/j.fochx.2025.102703

**Published:** 2025-06-25

**Authors:** Hany S. El-Mesery, Abdulaziz Nuhu Jibril, Ahmed H. ElMesiry, Zicheng Hu, Xinai Zhang, Amer Ali Mahdi

**Affiliations:** a School of Energy and Power Engineering, Jiangsu University, Zhenjiang 212013, China; b Agricultural Engineering Research Institute, Agricultural Research Center, Dokki, 12611 Giza, Egypt; c College of Engineering, Nanjing Agricultural University, Nanjing 210031, China; d Faculty of Computer Science and Engineering, New Mansoura University, 35742, Egypt; e School of Food and Biological Engineering, Jiangsu University, Zhenjiang 212013, China; f Department of Food Science and Nutrition, Faculty of Agriculture, Food, and Environment, Sana'a University, Sana'a, Yemen

**Keywords:** Microwave drying, Allicin content, Flavor strength, Principal component analysis, Artificial neural network

## Abstract

This study evaluates the physicochemical characteristics of garlic slices dried using a microwave-assisted convective dryer controlled by an artificial neural network. The chosen drying conditions included: microwave power (100, 200, and 300 W), air temperatures (45, 55, and 65 °C), and airflow velocity (0.3, 0.5, and 1.0 m/s). Results showed that at 65 °C, 300 W, and 0.3 m/s, the minimum flavor was 4.95 mg/g dry mass, marking a 39.50 % reduction in allicin content. The highest vitamin C content of 0.1751 mg/g with a water activity level of 0.505 was recorded at drying conditions of 1.0 m/s, 45 °C, and 100 W. However, it was observed that increasing power to 300 W at 45 °C and 0.5 m/s improved the rehydration ratio by 15.53 %. This study utilized precise ANN modelling to achieve an excellent fit by clarifying the interactions among drying parameters, time, and physicochemical parameters. PCA highlighted notable similarities between total color changes and rehydration ratios of garlic samples. Integrating an ANN into microwave-convective drying provides advanced tools to optimize food drying processes, thereby enhancing productivity without compromising product quality.

## Highlights

• Machine learning offers innovative and reliable solutions for addressing food spoilage.
• Microwave drying has gained recognition as an efficient alternative for drying food.
• The drying time increased with airflow but decreased with microwave power.
• The ANN proved a robust tool for predicting and optimizing garlic's physicochemical properties.

## Graphical abstract

**Figure:**
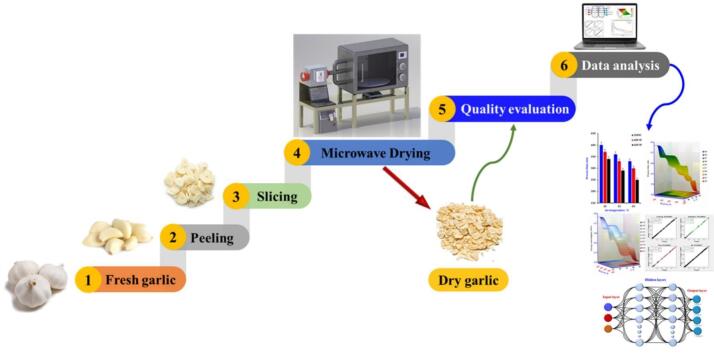

## 1 Introduction

Garlic can exhibit a wide range of biological effects due to its rich phytochemical composition, which mostly consists of ajoene, allicin, and sulfur-containing chemicals The health benefits of the bioactive components include antioxidant activity, cardiovascular support, anti-inflammatory effects, immune system enhancement, anticancer potentials, antiviral activity, digestive benefits, improving bone health, detoxification support, antiviral, antibacterial, and antifungal properties. Fresh garlic deteriorates easily due to its high-water content (more than 75 %), which shortens its shelf life and results in financial losses (Feng et al., 2020; Huang, Ding, Zhao, Li & Ma, 2018). Efficient processing methods are needed to increase its shelf life and preserve nutritional value. Food can be dried to eliminate 90 % of its water content, which also reduces water-mediated degradation reactions, lowers transportation expenses, and reduces the spoilage caused by microbial development. However, drying techniques have been categorized into non-thermal and thermal processes. Microwave drying has both thermal and nonthermal effects, while hot air drying and infrared drying are the primary thermal drying methods. Consequently, the quality of the intended food is affected differently by different dehydration methods (El-Mesery et al. 2024a; Zhou et al., 2021).

Microwave drying is an innovative and promising technique that offers several advantages over traditional hot-air drying methods, including faster drying rates, reduced water accumulation, and a minimized risk of overheating. These benefits contribute to improved preservation of the functional qualities of the product, which is particularly important in industries like food and pharmaceuticals (Sellami et al., 2011). However, for microwave drying to be more effective at the industrial scale, there are still challenges to overcome, such as non-uniform temperature distribution and limited penetration depth in dense materials. These issues can lead to potential degradation, especially for heat-sensitive components of products like garlic slices. To address these limitations, recent studies have explored combining microwave drying with other dehydration methods, such as hot air drying, to enhance the overall process. For example, microwave-hot air drying has shown promise by combining the advantages of both techniques, thereby reducing the drawbacks of each method and improving product quality (Osae et al., 2020). This approach has been successfully applied to various food products, including herbs like cannabis and stevia leaves, as well as vegetables like cherry tomatoes and lentils, where the goal is to preserve the shelf life and quality of the products while overcoming the inherent limitations of each drying method (Das, Baik, & Tabil, 2024; Lemus-Mondaca, Zura-Bravo, Ah-Hen &Di Scala, 2021; Najib, Heydari, & Meda, 2022). Ultimately, selecting and refining the best dehydration technique for each product to preserve its functional properties as closely as possible to those of the original items (Jibril et al., 2025).

Machine learning models are now essential for precisely forecasting and improving engineering systems, providing a useful substitute for energy efficiency and quality enhancement in the drying operation. The ANN model predicts the complex outcomes of the drying system using the data generated. Recently, it is essential to use artificial neural networks to study numerous interactions in different fields, particularly food processing (Jibril et al., 2024). The artificial neural networks (ANN) are widely used in comparison to other conventional modelling techniques due to their easy operations, forecasting power capabilities, and flexibility in reducing or increasing input and output variables (El-Mesery, et al. 2025a). Machine Learning is a novel technique that uses experimental data and tries to induce the best non-linear or linear model instead of requiring knowledge of a specific physical relationship between input and target data. This model helps when it is incredibly challenging to identify an analytical model that best captures the relationship, and there are very non-linear relationships between the input and target data. Artificial neural networks are widely recognized as the most popular tools to simulate and optimize intricate drying processes. This computational method examines the connection between output responses and input parameters using sparse experimental data.

ANN is a potent tool for outcome prediction because of its ability to learn from prior data and generalize complex nonlinear process behaviours. However, it's increasingly considered a strong and acceptable substitute for process modelling and optimization. Recently, Marić et al. (Marić et al., 2020) used ANN to forecast root vegetables' physical and chemical properties, following traditional drying, and artificial neural networks demonstrated R^2^ of over 80 %. Kırbaş et al., (Kırbaş, Tuncer, Şirin, & Usta, 2019) revealed that with R values exceeding 99 %, ANN was effectively used to forecast pomelo fruit's online moisture content change using various drying techniques, such as microwave, freeze-drying, and forced convection. In food processing, machine learning has been used extensively for drying process optimization and modelling. Several machine-learning models, such as ANN, SVR, and RF, have been used to reduce energy usage, forecast drying times, and evaluate the quality of the finished product. Real-time food processing monitoring and control using machine learning has also been investigated to improve quality and energy efficiency. Numerous studies rely on a small dataset and basic models that are unable to capture the basic interactions taking place in drying, despite the recent breakthroughs in machine learning. Furthermore, it is still difficult to optimize numerous variables at once because the majority of machine learning (ML) models are not incorporated into real-time systems intended for adaptive control (Miraei Ashtiani & Martynenko, 2024).

Nevertheless, Machine learning modelling is a crucial technique for producing higher-quality products and optimizing the drying process. Moreover, no research has been done to date to forecast the physicochemical features of garlic slices of the unique MW-HA drying method, although ANNs have been extensively studied for predicting various drying processes. Therefore, this study investigates the impact of different velocities, temperatures, and microwave power on the hybrid microwave-convective dryer. It further evaluates its effects on the physicochemical characteristics of garlic slices, such as their overall color change, vitamin C, flavor intensity, water activity, rehydration ratio, and allicin content, during the hybrid microwave-convective drying process. It develops a model based on machine learning to predict how the physicochemical quality of dried garlic slices would vary over time. It evaluates the application of principal component analysis (PCA) to understand the interactions between the physicochemical characteristics of dried garlic slices and drying conditions.

## 2 Materials and methods

### 2.1 Material

Fresh garlic bulbs were being purchased from a farm in Xuzhou (Jiangsu, China), and it was stored at 4 ± 1 °C in the refrigerator before the experiment. Garlic cloves were immersed in a sodium hypochlorite solution (200 ppm) for 2 min to reduce microbial load, then rinsed with sterile distilled water to remove any residual chlorine. An industrial food slicer (SS-250, SEP Machinery Company Ltd., Guangzhou, China) was used to peel the cloves and slice the garlic to a 5 mm thickness.

### 2.2 Microwave convective hot air dryer

**Fig. 1:**
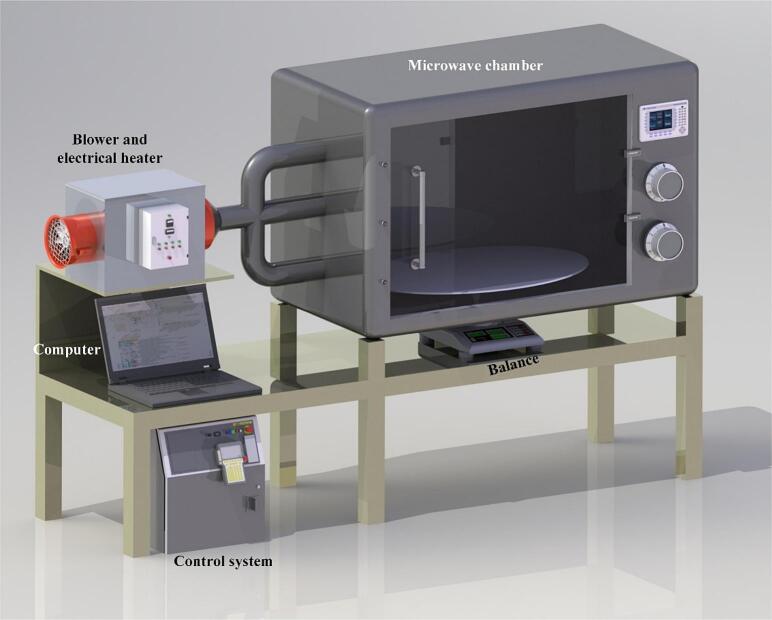
Schematic diagram of microwave convection hot air dryer.

The drying procedures were conducted using a novel combination microwave dryer (Fig. 1). The hybrid dryer facilitates both convective and microwave drying, allowing for separate operations and various combinations of the two methods. The air heating system consists of an electric heater and fan that provides hot air for convective drying. Two spiral electrical heaters were used to heat the air. The microwave oven includes Panasonic's microwave oven (NN-CD997S) with dimensions of 55.5 × 68.5 × 43 cm, with a power of 1000 W. The microwave oven controls the power to 100, 200, 300, 400, 600, 800, and 1000 W. The dimensions of the dry space are 462 mm, 242 mm, and 412 mm, featuring at the bottom of the oven with a revolving glass plate of 380 mm. The generated power and execution time of the microwave were entirely controlled via the digital control panel of the microwave oven. The heated air flows across the wall opposite the entrance after passing through the plenum microwave oven. This enabled hot air to circulate beneath the drying pan and over the drying slices. The airflow was evaluated by an anemometer (± 0.1 m/s). PT100 temperature sensors (± 0.1 °C) are fitted to this dryer's electrical heating unit, which is connected to a data logger.

### 2.3 Drying approaches

Three levels of microwave power (100, 200, and 300 W), air temperatures (45, 55, and 65 °C), and velocity (0.3, 0.5, and 1.0 m/s) were utilized under a hybrid microwave drying system. Each test positioned a sample of 300 g of garlic slices in the hybrid dryer. The garlic slices' weight changes were monitored using digital load cell systems with a precision of 0.01 g, installed at the base of the setup.

### 2.4 Moisture content

A 10 g garlic sample determined the initial mass (Mi). The final mass (Mf) was calculated after drying the sample in an oven at 105 °C until a consistent mass was achieved (AOAC, 2005). However, the initial moisture content of the garlic sample is 67.8 ± 0.8 % (w.b.). The final moisture content was determined using the formula in Eq. 1.

(1) $$\text{Moisture content}\;\left({\mathrm{kg}}_{\text{water}}/{\mathrm{kg}}_{\mathrm{dry}\;\text{matter}}\right)=\frac{M_{i}-M_{f}}{M_{f}}\;$$

The slice's mass was logged automatically at 5-min intermissions, allowing Eq. 2 to estimate its moisture content at drying periods (MCt).

(2) $${\mathrm{MC}}_{t}=\frac{M_{t}-M_{o}\;\left(1-{\mathrm{MC}}_{o}\right)}{M_{o}\;\left(1-{\mathrm{MC}}_{o}\right)}$$

Fick's law mathematical model was used to determine the moisture ratio as a function of the drying time.

### 2.5 Water activity

3 g of garlic slices were used in the LabMASTER technique to measure water activity (a_w_) at 25 °C. The device accurately and consistently monitors water activity (a_w_) by enclosing the garlic slices in a closed, controlled chamber to measure the air humidity.

### 2.6 Vitamin C

The 2,6-dichlorophenol indophenol standard titration method was used to measure the vitamin C content of the garlic slices. The vitamin C content evaluation was conducted in three replicates.

### 2.7 Rehydration ratio

The rehydration properties of dried garlic slices were determined by immersing them in boiling water. The dried slices (10 g) were field hooked on a beaker containing 150 ml of filtered water, and the boiling process continued for five minutes while keeping the glass sealed (El-Mesery et al., 2023a). The dried sample's rehydration ratio (Rr) was calculated using Eq. 3.

(3) $$\mathrm{RR}=\frac{W_{r}}{W_{d}}$$

Wd and Wr are the weight and rehydrated of the dried garlic slices (g).

### 2.8 Color analysis

A colorimeter was used to measure the color of both fresh and dried samples using the procedure described by (Bai, Xiao, Le Ma, & Zhou, 2018). The sample's surface was used to extract the five color capacities for every analysis, and the average of the color changes was evaluated. The measurement head was calibrated using a white calibration plate. The fresh and dried product's parameters a* (red/green), b* (yellow/blue), and L* (brightness) were measured after standardization. The average results were recorded after ten replications for each sample. Eq. 4 was used to measure the overall color difference (*δ*E).

(4) $$\mathrm{\delta E}=\sqrt{{\left(L^{*}-L_{o}^{*}\right)}^{2}+{\left(a^{*}-a_{o}^{*}\right)}^{2}+{\left(b^{*}-b_{o}^{*}\right)}^{2}}\;$$

where L_0_*, a_0_*, and b_0_* represent the fresh garlic slices color parameters, and L*, a*, and b*, represent the dried garlic slices color parameters.

### 2.9 Allicin content (AC)

The procedure described by Feng et al. (Feng et al., 2019) was used to measure the allicin content in the fresh and dried garlic samples using spectrophotometric techniques at 412 nm. Eq. 5 was used to extract the allicin content as relative retention compared to fresh garlic.

(5) $$\mathrm{AC}\;\left(\%\right)=\frac{A_{t}}{A_{o}}\times 100\%$$

The fresh (mg/g) and dried (mg/g) samples' allicin contents are Ao and At, respectively.

### 2.10 Flavor content

The pungent sulfur-containing volatile oil of the garlic slices was evaluated using the Chloramine-T technique as described by Madhu et al. (Madhu, Mudgal, & Champawat, 2021). The volatile oil content in the garlic sample was calculated as milligrams of oil per gram of dry matter.

### 2.11 Machine learning approaches

The machine learning approach is widely used to solve engineering problems associated with classification and regression tasks. It provides predictions from the acquired knowledge in the learned dataset. This present study used an ANN model to forecast the drying time and physicochemical characteristics.

#### 2.11.1 Artificial neural network

ANNs are models that can draw inspiration from the brain's data processing capabilities, while learning complex associations from comparatively small datasets. These algorithms use simplicity to mimic the functioning of biological neural networks, which are found in the human brain. They consist of a neural network (ANN), which is an intricate structure of neurons with weights, interactions, output, hidden, and input layers. This design is commonly represented as a Multilayer Perceptron (MLP), which is composed of an output layer, one or more hidden layers, and an input layer. During the training, the experimental data were introduced into the input layer, and the network modified connections and weights throughout the layers to generate the intended outputs in the output layer (Arunkumar, Balaji, Madhankumar, & Mohanraj, 2024). The experimental data was modelled using WEKA software (v3.9.6, New Zealand) and split into three sets at random: 15 % for validation, 15 % for testing, and 70 % for training. The design model comprises three input factors (temperature, power, and velocity) and six output parameters (drying time, rehydration ratio, total color change, flavor strength, water activity, vitamin C, and allicin content). Eqs. (6) and (7) provide the broad output and error functions (E_r_) of an MLP.

(6) $$q_{i}=f\left(\sum_{i.j=1}^{l,m}\left(W_{\mathrm{ij}}.P_{i}\right)\right)$$

(7) $$E_{r}=\frac{1}{N}\sum_{i=1}^{N}{\left(E_{i}-q_{i}\right)}^{2}\;$$

Pi, f, E_i_, w_ij_, q_i_ represents the input data, activation function, expected output value, weight, and net output factor.

#### 2.11.2 Principal components analysis (PCA)

Principal components analysis (PCA) uses an orthogonal array approach to change the initial data set of potentially related parameters into a new data set of linearly uncorrelated parameters to decipher the complex network of connections and differences between the different quality indicators. Advanced PCA analysis tools were used to evaluate the relationship between the water diffusion parameters and energy efficiency with OriginPro 2024SR1, version 10.1.0.178 (Massachusetts, USA).

#### 2.11.3 Model performance

The sum of square errors (SSE), correlation coefficient (R^2^), and root mean square error (RMSE) were used to evaluate the model's performance. The optimal model for predicting the anticipated parameter of dried garlic was determined to be the one with the least SSE, the highest R2 value, and the lowest RMSE (El-Mesery et al., 2023b).

(8) $$R^{2}=\frac{\sum_{i=1}^{N}\left(y_{i}-x_{i}\right)}{\sum_{i=1}^{N}{\left(y_{i}-x_{i}\right)}^{2}}$$

(9) $$\text{RMSE}=\sqrt{\frac{1}{N}\sum_{i=1}^{N}{\left(x_{i}-y_{i}\right)}^{2}}$$

(10) $$\mathrm{SSE}=\sum_{i=1}^{N}{\left(x_{i}-y_{i}\right)}^{2}$$

yi, and x_i_ represent the predicted and observed parameters, while N is the number of observations.

### 2.12 Statistical analysis

Sophisticated analysis tools, such as SPSS 2022, version 27.0.1.0 (Chicago, USA), were used to analyse the experimental parameters on three-way ANOVA. The Duncan test groups the experimental values at a 5 % significance level. Each drying condition was performed in triplicate. Principal components analysis (PCA) uses an orthogonal array approach to change the initial data set of potentially related parameters into a new data set of linearly uncorrelated parameters to decipher the complex network of connections and differences between the different quality indicators. Advanced PCA analysis tools were used to evaluate the relationship between the water diffusion parameters and energy efficiency with OriginPro 2024SR1, version 10.1.0.178 (Massachusetts, USA).

The correlation coefficient (R^2^), sum of square errors (SSE), and root mean square error (RMSE) were computed to assess the fit quality of each mathematical model that was fitted to the moisture ratio curves over time for various drying circumstances. The best model that fits the data had a minimum SSE, the lowest RMSE values, and a better R^2^ value closer to 1. However, a new model was established after choosing the model with the best fitting prediction by incorporating variables such as microwave power, velocity, and temperature into the selected model.

## 3 Results and discussion

### 3.1 Drying time

**Fig. 2:**
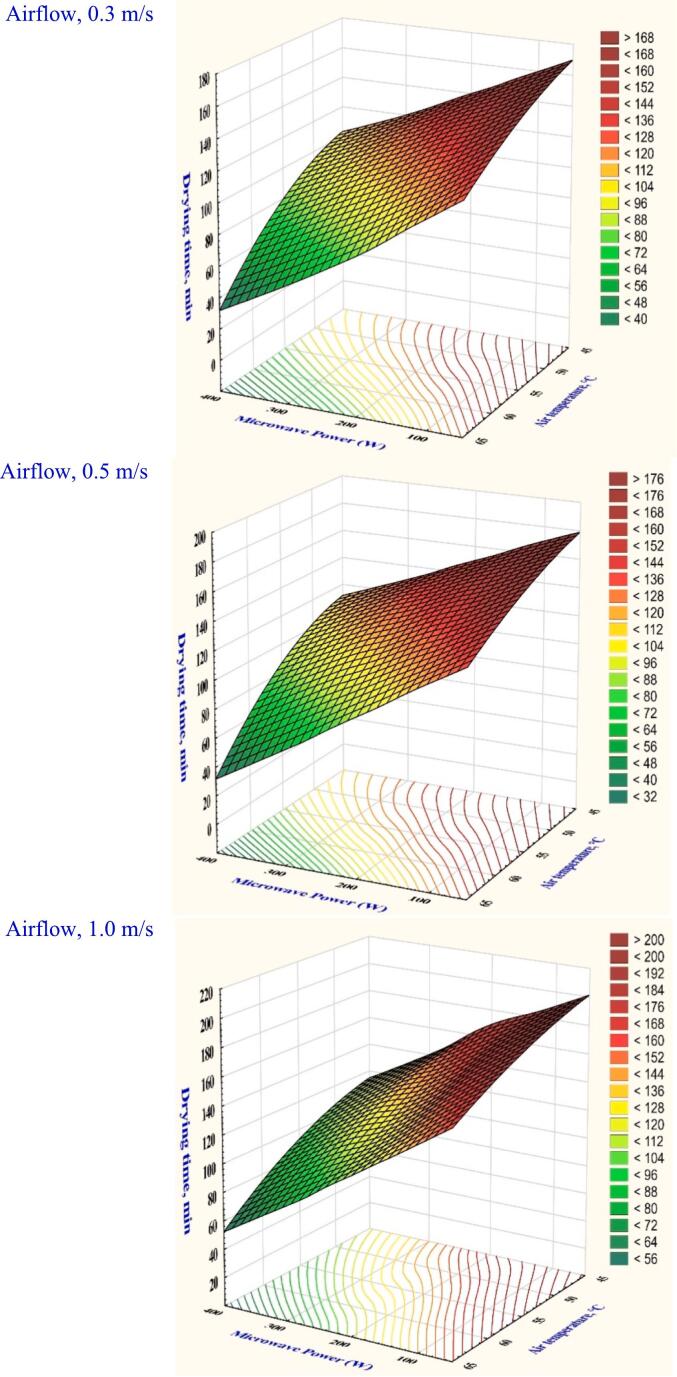
The variation in microwave power and air temperature affects the drying time of garlic slices when dried in a hybrid microwave dryer with different airflow rates.

The hybrid of microwave-convective (MW-HA) drying system was used to dry garlic slices at various powers (100, 200, and 300 W), air temperatures (45, 55, and 65 °C), and airflow velocities (0.3, 0.5, and 1.0 m/s). However, Fig. 2 demonstrates that a decrease in drying time was driven by increased microwave power. It was found that, with drying parameters of 45 °C, microwave power of 100 W, and velocity of 1.0 m/s, the peak drying time was measured at 190 min. As a result, at 300 W, a significant 35.71 % drying time decrease was noted while keeping the air temperature and velocity constant. This drop resulted from the vapor pressure difference between the product's exterior and interior, which was caused by the penetration of microwave energy. However, the drying process is accelerated by concentration gradient, increasing the microwave power, and the moisture diffusivity (El-Mesery & Elabd, 2021).

Fig. 1 illustrates that drying time was also affected by the air velocity. It was shown that longer drying time was achieved by increasing airflow at a specific temperature and microwave power. The highest drying time for garlic slices was 160 min at a velocity of 0.3 m/s, microwave power of 100 W, and temperature of 45 °C. This drying time rose dramatically to 190 min by increasing the velocity to 1.0 m/s. This was attributed to the phenomenon of the product surface drying. However, higher velocity would cause the product's surface temperature to drop, which would reduce the moisture diffusivity (Khoshtaghaza, Darvishi, & Minaei, 2015).

### 3.2 Rehydration ratio

**Fig. 3:**
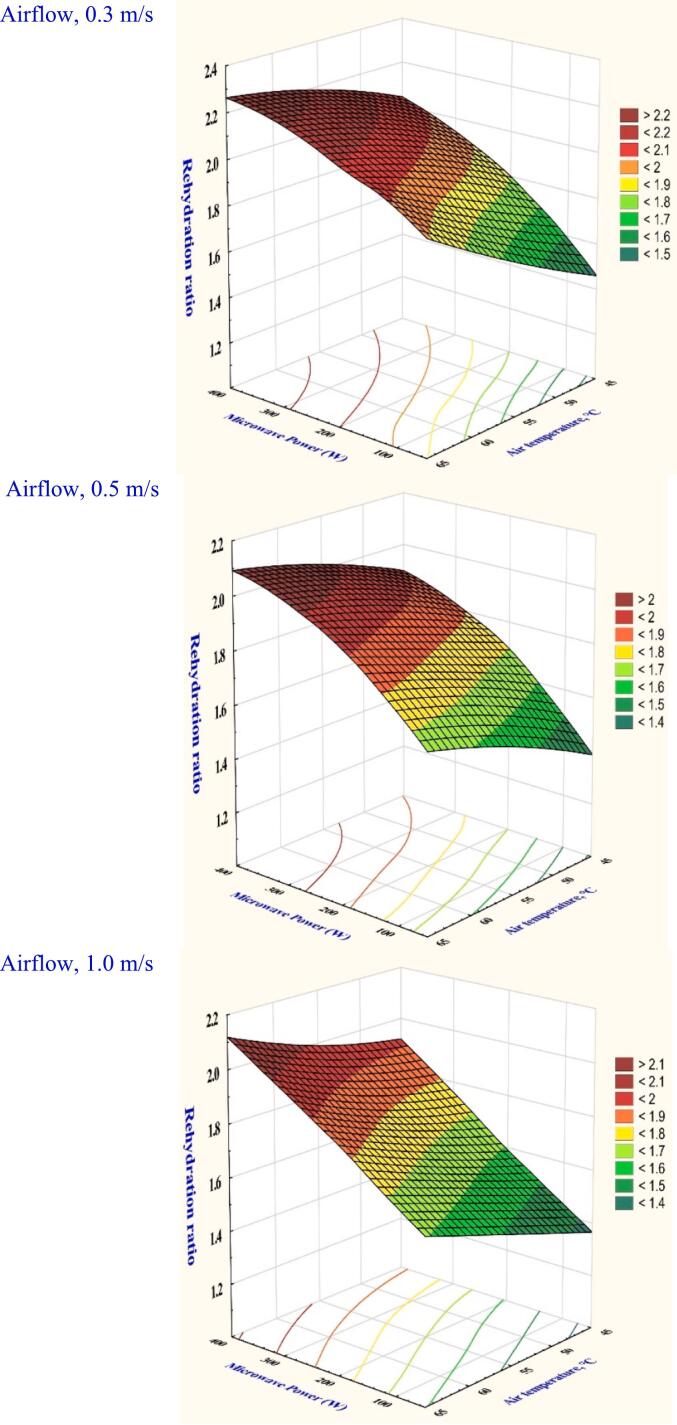
The impact of drying conditions on the rehydration ratio of garlic dried in a hybrid microwave dryer.

Rehydration reflects on the internal microstructure damage and shows the material's capacity to reabsorb moisture after drying. Table S1 presents the analysis of variance conducted under different drying settings showed significant effects (*p* < 0.05) on the rehydration ratio. The MW-HA drying rehydration values were determined to be between 1.48 and 2.17 at varying combinations of drying settings in Table S1. However, the lowest rehydration ratio value was recorded at 1.0 m/s of velocity, 45 °C temperature, and 100 W of microwave power. Nevertheless, the highest value was noted when a higher power of 300 W, a maximum of 65 °C temperature, and 0.3 m/s of velocity were used. Drying at a higher microwave power increases the vaporization of water, internal heating, and pressure gradient through greater microwave absorption. Accordingly, the hybrid microwave dryer for the garlic sample caused less harm to the cell's interior structure than HAD and the infrared dryer described by EL-Mesery et al. (EL-Mesery, Sarpong, Xu & Elabd, 2022). The impact of temperature and microwave power resulted in changes in garlic rehydration properties. Fig. 3 illustrates that the dried garlic slice a constant 0.5 m/s of velocity and 45 °C of temperature recorded an increase in rehydration ratio from 1.61 to 1.86 with a rise in microwave power from 100 to 300 W. This was attributed to the fact that increasing the rate of absorption in microwave caused the water in the garlic slices to evaporate faster, preventing the shrinkage that improved the product's hardening. Kumar & Shrivastava (Kumar & Shrivastava, 2017) reported that the rehydration ratio increased from 6.25 to 10.75 when the vacuum was increased from 200 mmHg to 600 mmHg and the microwave power was increased from 100 to 300 W for green bell peppers.

### 3.3 Color changes

**Fig. 4:**
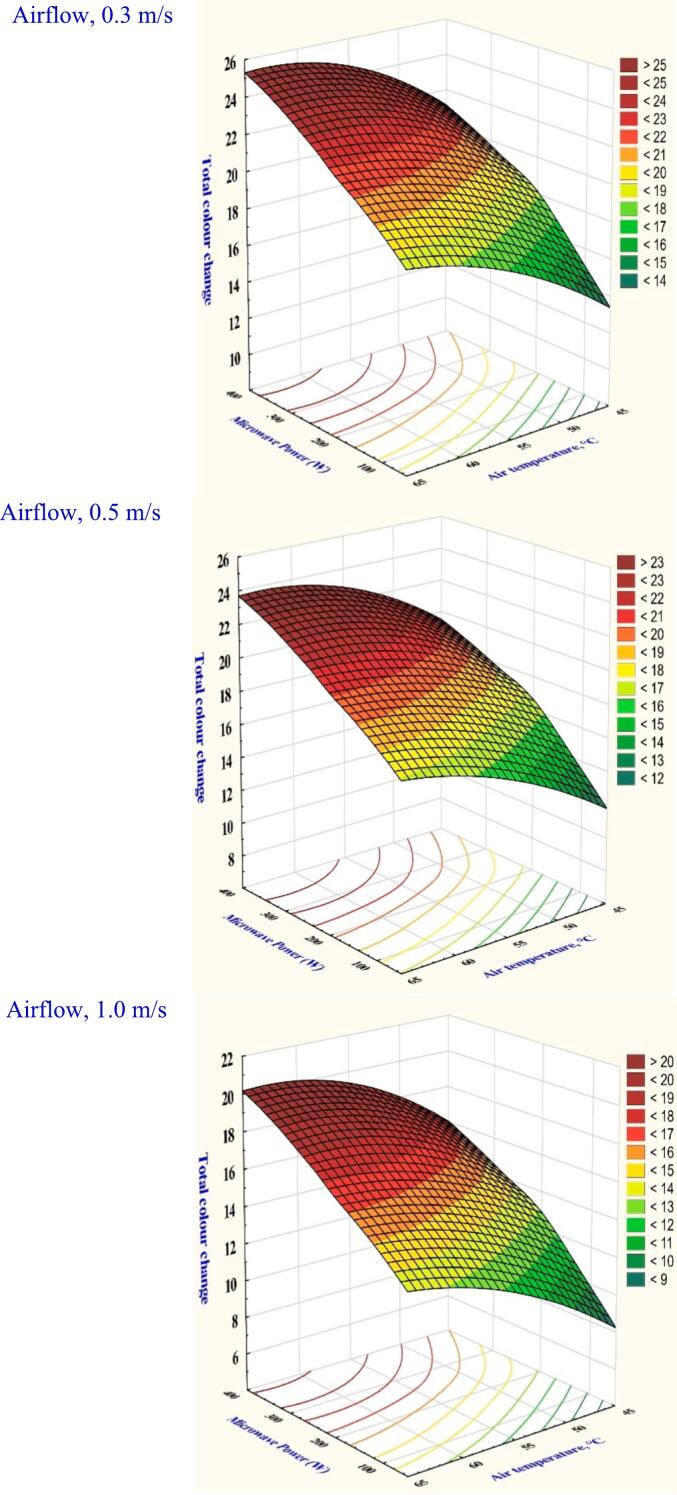
The effect of drying conditions on the overall color changes of garlic dried in a hybrid microwave dryer.

Color variation is a crucial element influencing consumer acceptability of a dried product. It's frequently used to assess the dried product quality attribute. A slight color adjustment can improve the dried product's added value and characteristics. Table S1 and Fig. 4 display the color variations between the dried garlic slices under various microwave-assisted hot-air drying settings, revealing that all variables analyzed had significant effects (*p* < 0.05). Fig. 4 shows that the maximum color change was 23.79 when drying conditions were set at 65 °C, 300 W of microwave power, and 0.3 m/s of velocity. The fastest drying cycle resulted from a lower browning reaction due to the greater drying temperature compared to the other drying parameters. Selvi et al. (Selvi et al., 2020) discovered that the rose petals observed lower color change at 70 °C compared to those at 50 °C and 60 °C. Nonetheless, Table S1 shows that the minimum color change was 23.79 when the drying conditions were at a microwave power of 100 W, a temperature of 45 °C, and a velocity of 1.0 m/s. Higher air velocity has been shown to accelerate the browning reaction of garlic samples and increase color change by causing non-enzymatic Maillard reactions that degrade pigment and cause browning. Furthermore, the color change increased to 14.45 % when maintaining the velocity at 0.5 m/s and microwave power at 200 W while increasing the temperature from 45 to 55 °C. This demonstrated that heating garlic slices in a microwave could deactivate the enzymes responsible for browning, minimizing the dried product's color change.

### 3.4 Flavor strength

**Fig. 5:**
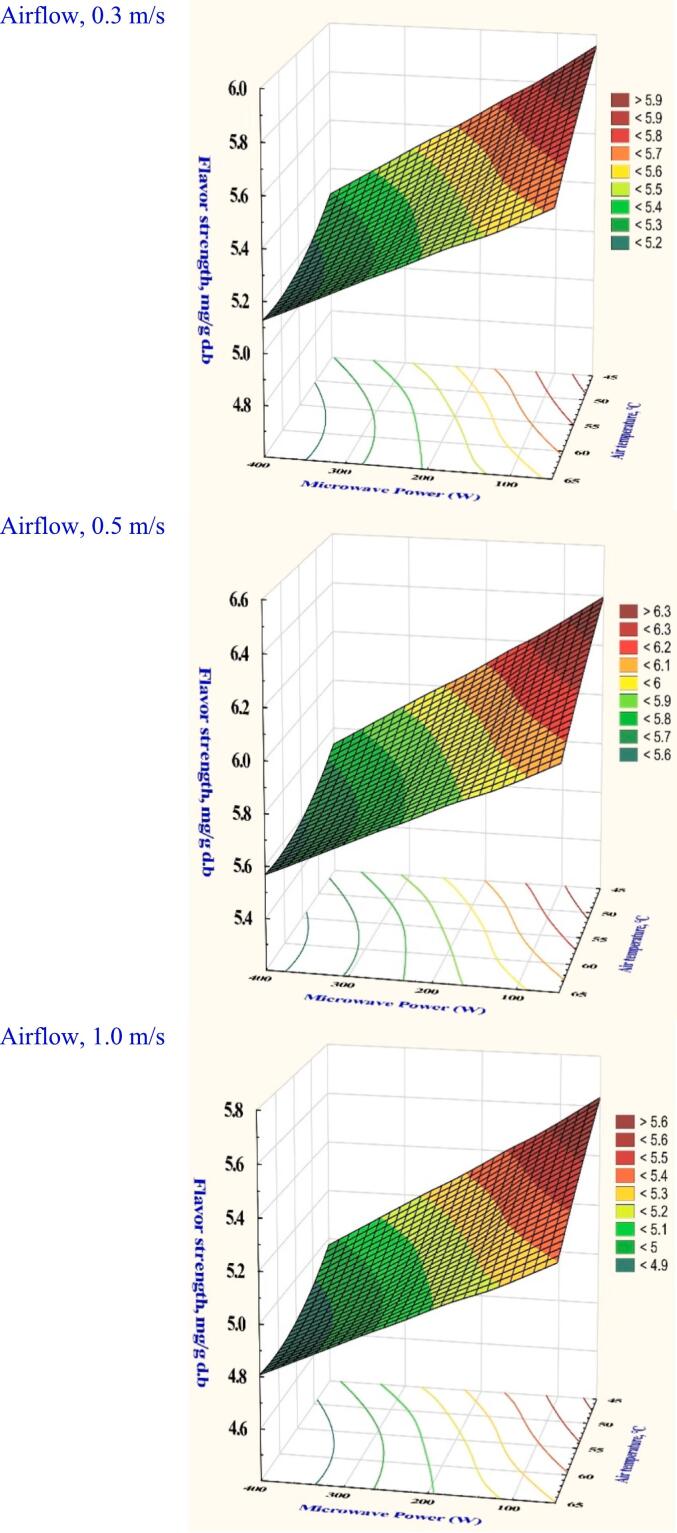
The effect of drying conditions on the flavor strength of garlic dried in a hybrid microwave dryer.

Flavor is an essential component of dried garlic slice quality that impacts customer acceptance and preferences. Table S1 presents the range of dried garlic slices' flavor content under various drying circumstances, indicating that the analysis of variance showed that all the variables examined had significant effects (*P* < 0.05). However, Fig. 5 presents a reduction in flavor intensity associated with average percentages of 13.74 % at 0.3 m/s, 10.31 % at 0.5 m/s, and 7.05 % at 1.0 m/s while increasing temperature from 45 to 65 °C, and a minimum power of 100 W. The average concentrations of 5.18 (mg/g dry mass) at 0.3 m/s, 5.50 (mg/g dry mass) at 0.5 m/s, and 5.94 (mg/g dry mass) at 1.0 m/s while maintaining power 200 W and air temperature 55 °C revealed that garlic flavor strength was shown to be enhanced by increasing the velocity. However, the lowest flavor content (4.95 mg/g dry mass) was observed under the drying condition of 65 °C, 300 W, and 0.3 m/s. This result can be attributed to thermal degradation of volatile sulfur compounds, which are highly sensitive to heat and microwave intensity. At 65 °C and 300 W, the combination of high thermal load and microwave energy likely caused rapid heating and excessive volatilization or breakdown of flavor compounds such as diallyl disulfide and allyl methyl sulfide. Furthermore, the low air velocity (0.3 m/s) likely limited the removal of moisture and volatiles efficiently, prolonging exposure to heat and promoting further degradation or loss of aroma compounds. This suggests that while moderate power and airflow can preserve flavor, the combination of high temperature, high power, and low velocity is detrimental to garlic flavor retention (El-Mesery et al., 2025b). The flavor profile of garlic components when subjected to catalytic infrared drying processes was highlighted by (Figiel, 2009), demonstrating how sensitive flavor components of garlic decreased after microwave drying.

### 3.5 Allicin profile

**Fig. 6:**
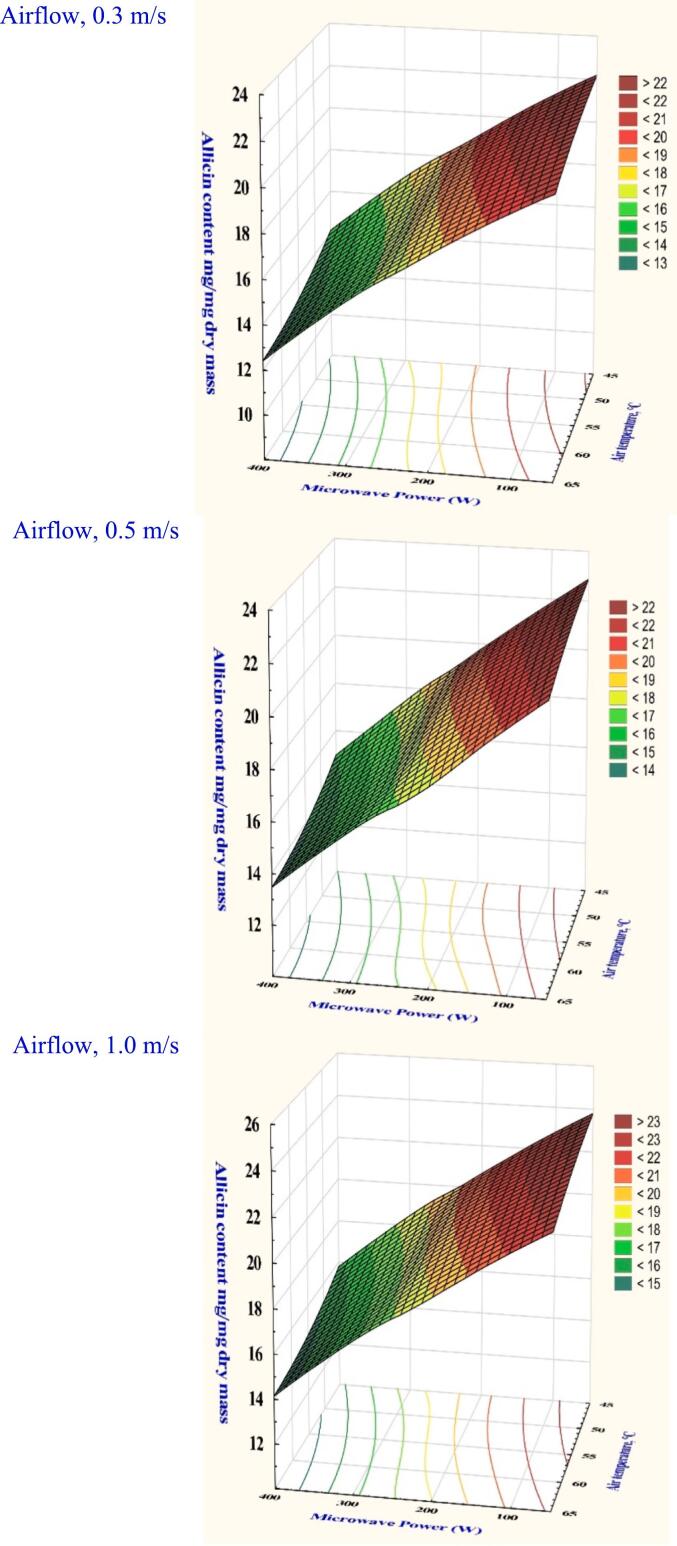
The effect of drying conditions on the allicin content of garlic dried in a hybrid microwave dryer.

Allicin content is an essential bioactive ingredient in garlic that significantly affects the flavor of garlic slices. Alliin and alliinase react in the cytoplasm to produce allicin. Allicin content is significantly impacted by the drying time and temperature, as presented in Fig. 6 and Table S1. The analysis of variance showed that all the variables examined had significant effects (*p* < 0.05). Having a velocity setting of 0.3 m/s and a temperature of 45 °C, the allicin percentages were 21.26 at 100 W, 19.11 at 200 W, and 16.90 at 300 W. In contrast to the lowest drying settings, the allicin concentration decreased significantly by 39.50 % when the air temperature was raised to 65 °C, while the microwave power remained at 300 W and the velocity was 0.3 m/s. This pattern shows that allicin levels decrease when power and temperature rise, regardless of airflow velocity. According to (Thakur, Dhiman, Kumar, & Gautam, 2025), the microwave dryer had better retention of the allicin components of garlic powder under improved microwave power of 180 W. (Calin-Sanchez, Figiel, Wojdyło, Szarycz, & Carbonell-Barrachina, 2014) reported that the presence of allicin and its derived products at higher microwave power could be responsible for the high antioxidant capacity of dried garlic slices.

### 3.6 Vitamin C

**Fig. 7:**
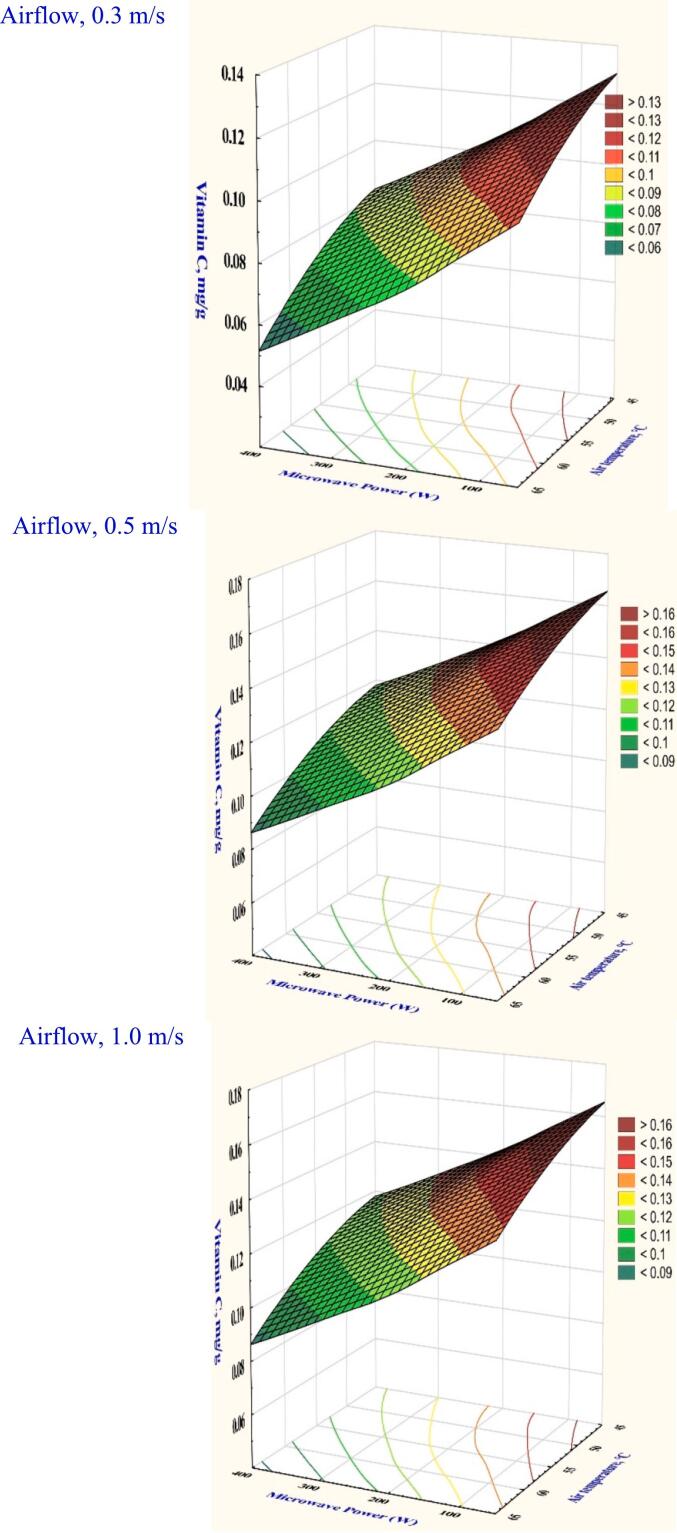
The effect of drying conditions on the Vitamin C content of garlic dried in a hybrid microwave dryer.

Vitamin C is a micronutrient abundant in garlic and important for human health. However, the amount of vitamin C decreases when food products are heated. The dry garlic sample recorded the lowest amount of vitamin C retention of 0.0701 mg/g at power 300 W, air temperature 55 °C, and velocity 0.3 m/s, as illustrated in Table S1. All the variables that were evaluated had significant effects, as indicated by the analysis of variance (*p* < 0.05). Furthermore, Fig. 7 shows that the vitamin C retention was significantly improved by 6.17 % at microwave power 300 W, temperature of 65 °C, and velocity of 1.0 m/s. This is attributed to higher heating power, which reduces the amount of vitamin C retention. At the lowest airflow velocity, the vitamin C profile increased from 0.0787 to 0.1231 mg/g when the air temperature was raised from 45 to 65 0C at a power of 100 W to 300 W. The increase in vitamin C to 0.1540 mg/g appeared to be dominated by exposing the garlic slice to a higher airflow velocity of 1.0 m/s, which resulted in a protracted minimum exposure to microwave power and temperature. Vitamin C is fragile and readily impacted by oxygen and extended temperatures. It was observed that when ultrasound was used to improve far-infrared radiation drying on pear slices, lower ultrasound powers were more advantageous in preserving vitamin C. It was explained that ultrasound could increase the degradation of the vitamin C profile compared to air-dried cabbages (Liu et al., 2019).

### 3.7 Water activity

**Fig. 8:**
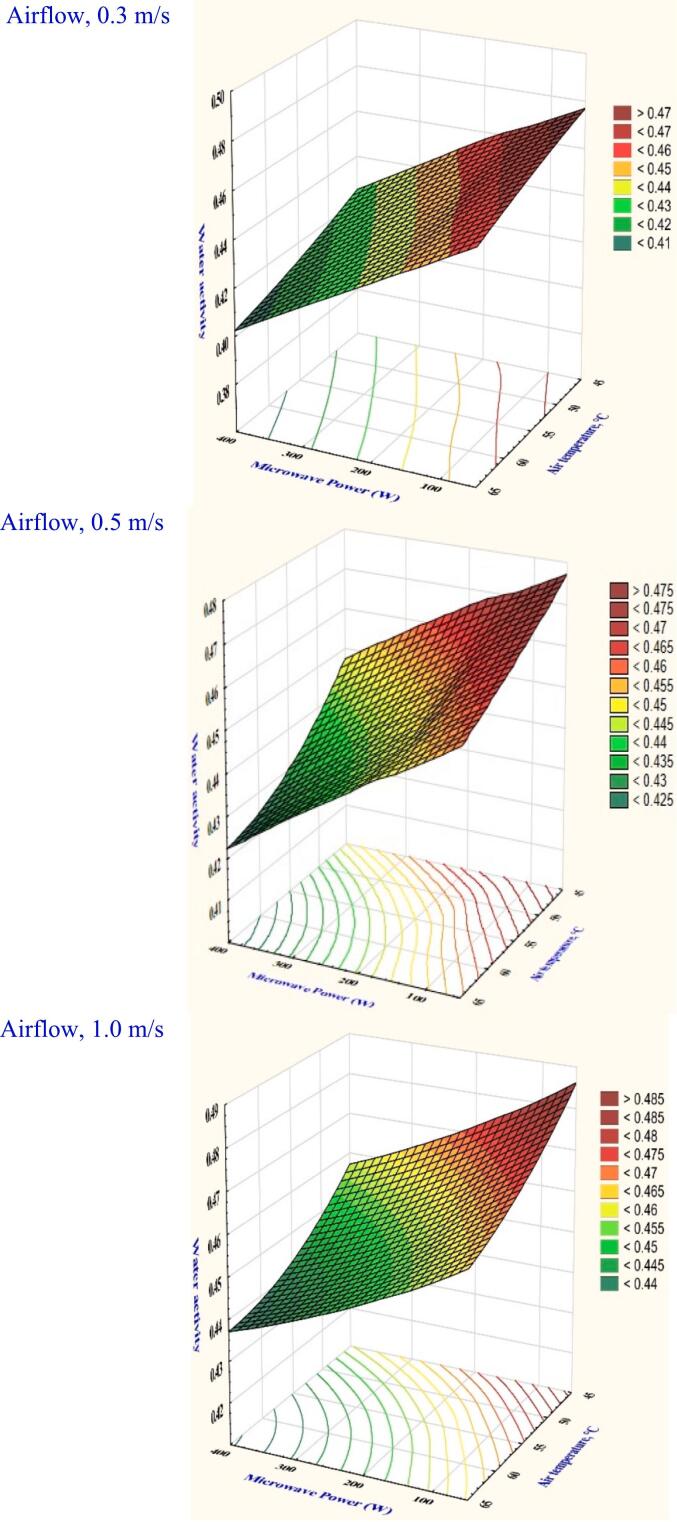
The effect of drying conditions on the water activity of garlic dried in a hybrid microwave dryer.

The water activity of a food product indicates its level of attraction for water. Water activity is considered low when food is classified as having a water level of less than 0.8, similar to the typical basic for growing bacteria and yeast. Table S1 indicates that the analysis of variance showed that all the variables examined had significant effects (*P* < 0.05). The peak water activity was recorded at 0.505 under drying parameters with a velocity of 1.0 m/s, a temperature of 45 °C, and a microwave power of 100 W. Consequently, maintaining the same temperature and microwave power recorded a considerable reduction in water activity at 0.5 m/s with 4.12 %, and at 0.3 m/s with 7.45 %. Moreover, water activity was significantly decreased by elevating the air temperature. Fig. 8 shows that at a maximum of 300 W of microwave power, 65 °C of temperature, and 1.0 m/s of velocity, the water activity is recorded as 0.455. This effect shows that hybrid microwave drying methods can successfully attain the required water activity for prolonged storage. (Bozkır, Ergün, Taştan, & Baysal, 2018) elaborated that increasing microwave power and decreasing airflow cause more water to evaporate off the surfaces of garlic slices. The necessary water activity can be achieved because of the aggressive water diffusion from the slices to the surface.

### 3.8 Machine learning approaches

#### 3.8.1 Analysis of artificial neural network prediction

ANN's model was used to forecast numerous characteristics of garlic slices drying processes. The models' input parameters were microwave power, airflow velocity, and temperature. At the same time, the output variables were allicin content, drying time, water activity, rehydration ratio, flavor strength, color change, and vitamin C retention. The influence of many learning methods, neurons, and function types on the model's forecast was investigated by designing and evaluating several network configurations. A trial-and-error method was used to select and simulate these network architectures. However, the configuration was achieved using one hidden layer due to the better prediction accuracy. The results of experimental and predicted values under different drying circumstances were compared to determine the goodness of the ANN architecture. Table S2 presents the results of training, validating, and testing the multi-layer perceptron model for drying time garlic slices. The optimal ANN for characterizing the garlic slices was determined using the drying time testing data set. As a result, the drying processes achieved the best prediction under different drying conditions of 45 °C, 1.0 m/s, and 100 W with a configuration of 3–15-1. The drying process for various biomaterials was shown to be highly predictable and to have an appropriate prediction of the capacity for artificial neural networks. ANN models were used by Momenzadeh et al. (Momenzadeh et al., 2011) to predict the drying time, and the results had the highest R^2^ prediction values at 98 % for green pea in a microwave-assisted fluidized bed dryer. However, El-Mesery et al. (El-Mesery et al., 2024b) observed that the artificial neural network model can accurately forecast garlic slices' drying period in a hybrid infrared dryer.

Results of training, validating, and testing artificial neural networks for garlic samples at different drying settings are displayed in Table S3. The objective was to identify the optimal model that could effectively predict the rehydration ratio for garlic slices based on the experimental data using the MW-HA dryer. The best projected model configuration of 3–12-1 was obtained at 200 W, 0.5 m/s, and 55 °C, yielding the lowest RMSE (0.0005–0.0010) and the highest R^2^ (0.9999–1.0000). El-Mesiry, et al. (El-Mesery et al., 2025) investigated the most effective approach for predicting the okra rehydration ratio with artificial neural networks (ANN). The results show that an ANN algorithm estimated and optimized the optimal rehydration ratio of okra slices. Similarly, Nejatdarabi and Mohebbi (Nejatdarabi & Mohebbi, 2023) used MLR and ANN to forecast the rehydration process of mushroom powder in potato starch, corn starch, and hydration properties.

The projected performance for training, validation, and testing data was assessed using R^2^ and RMSE on total color change. It revealed that a robust model was designed when the R^2^ value is closer to 1 and the RMSE value is close to 0. The performance of the ANN models assessed by training, validation, and testing sets is shown in Table S4. It was noted that the best predicted model performed with the better testing accuracy of R^2^ = 1.0000, and a minimal RMSE value of 0.0106. Furthermore, the ANN demonstrated a centralized data distribution and a consistent prediction impact when dried at 300 W, 65 °C, and 0.3 m/s, with a configuration of 3–8-1. A similar result was reported when using a ML method to forecast the okra drying process based on color analysis (El-Mesery et al., 2025). The results show that radiation interaction was more significant for the predicted color behavior in ANN. Nadian et al. (Nadian, Rafiee, Aghbashlo, Hosseinpour, & Mohtasebi, 2015) report that ANN could accurately predict the color of apple slices in hot air drying, demonstrating a correlation value of more than 0.92.

Table S5 shows the multi-layer perceptron's trained, validation, and tested results for garlic slices under various drying circumstances. The training and the tested data were used to minimize the contradiction found in the tested data. The best ANN for characterizing the strength of garlic flavor was evaluated using a testing data set with a configuration of 3–8-1. The optimal drying conditions for flavor content were 65 °C, 300 W, and 0.3 m/s. The results were similar to the value reported for infrared drying of garlic slices with artificial neural networks (El-Mesery et al., 2025). The ANN model projected flavor changes using MSIR drying and MW-VC drying, and yielded regression performance with R^2^ values of 0.9418 and 0.9633, respectively.

The performance results of the artificial intelligence model for predicting the allicin content in garlic slices under various drying settings are displayed in Table S6. For configuration of 3–10-1 in testing, validation, and training, the best R^2^ values were 0.9999, 0.9998, and 0.9999 at 65 °C, 200 W, and 0.5 m/s, respectively. On the other hand, the least RMSE values were 0.0097, 0.0133, and 0.0075. Similar patterns were reported by El-Mesery et al. (El-Mesery et al., 2024) for evaluating allicin levels on garlic slices using an infrared dryer. The outcome demonstrates that ANN can forecast ideal drying conditions to balance allicin retention and time efficiency. According to El-Mesery et al. (El-Mesery et al., 2025), a SOM and artificial intelligence prediction model were used to optimize the physico-chemical characteristics of dried garlic slices. The outcome showed that the ANN can predict the allicin content with 99 % accuracy.

The predicted ANN models' statistical analysis values for the vitamin C profile of garlic slices under varied drying settings are displayed in Table S7. The predictive statistics of models with various sampling types and split ratios of training, validation, and testing demonstrated that a testing set RMSE of 0.0002 and R^2^ value of 0.9999 were much more accurate at 65 °C, 200 W, and 0.5 m/s than other drying conditions. This demonstrated that the distribution of datasets used in the model's training procedure for the garlic slices spanned a greater range of parameters than the other circumstances, having a configuration of 3–13-1. Chokphoemphun et al. (Chokphoemphun, Hongkong, & Chokphoemphun, 2024) observed similar results when using artificial neural networks (ANN) to assess garlic samples' behavior and drying properties under infrared convective heating. The results demonstrate the adaptability and dependability of the ANN model in forecasting the vitamin C characteristics of various fruits throughout the drying process. The effective use of ANN in numerous studies shows that it can be a valuable tool for streamlining drying processes and ensuring uniform product quality of fruits.

Table S8 shows that the ANN model performed better in predicting water content at drying conditions of 65 0C, 300 W, and 0.3 m/s, with the minimum RMSE value of 0.0001 and peak R^2^ value of 0.9999, and the model configuration was 3–9-1. It exhibits the poorest predictive performance at 55 0C, 100 W, and 1.0 m/s, having an RMSE of 0.0013 and R^2^ of 0.9902. Similar results about water content utilizing infrared heating were reported by ANN. The findings produced a precise forecasting model that closely matched the testing data sets, which provide new information for comprehending and managing the variables influencing the water content during the drying process of okra. ANN was used to predict the water activity of dried semi-finished cassava crackers, and the experimental outputs had MSE and R^2^ of 0.0034 and 0.9910, respectively (Lertworasirikul, 2008).

#### 3.8.2 Principal component analysis (PCA)

**Fig. 9:**
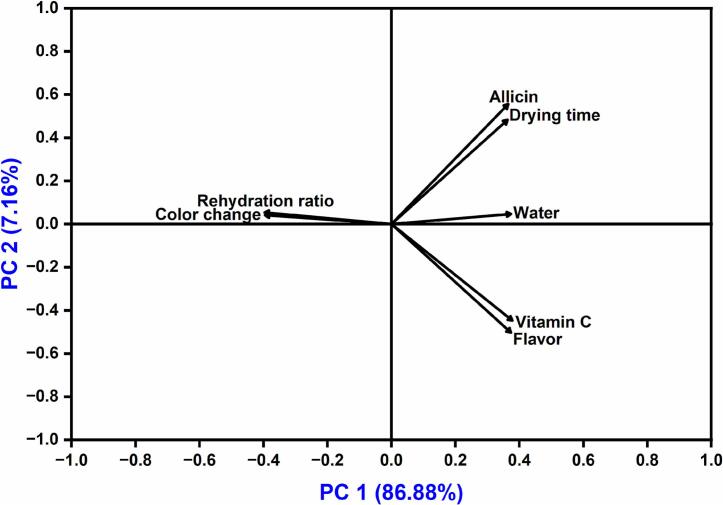
Principal component analysis (PCA) analysis on the interaction between drying time and physicochemical parameters.

**Fig. 10:**
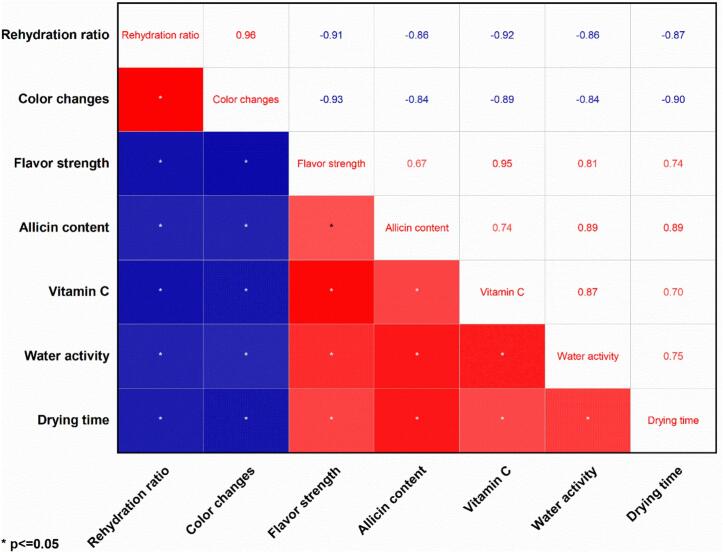
Correlation matrix on the interaction between drying time and physicochemical parameters.

PCA analyzes the connection between various variables from different observations (Karmakar et al., 2023). In the present study, PCA elucidated the correlation between the output parameters of drying time, rehydration ratio, total color change, flavor strength, water activity, vitamin C, and allicin content, which were analyzed depending on their interactions with the drying conditions. However, Fig. 9 shows that PC1 and PC2 represented 86.88 % and 7.16 % of the overall data variable, respectively. Therefore, the sum of variance explained by PC1 and PC2 was more than 80 % of the entire dataset, suggesting that the variables analyzed by PCA can better capture the data in the sample of garlic slices. Fig. 9 shows that the allicin, drying time, and water activity cluster on the positive side of both PC1 and PC2, with drying showing a significant positive correlation with both allicin content (*r* = 0.89) and water activity (*r* = 0.75) in Fig. 10. This suggests that longer drying times under certain conditions help preserve allicin and water activity, possibly due to milder drying conditions that reduce thermal degradation. In contrast, rehydration ratio and color change are located on the positive side of PC2, implying that samples with better rehydration properties tended to maintain more desirable correlation on color characteristics (*r* = 0.96), possibly due to minimal structural and pigment damage during drying. Vitamin C and flavor content also load positively on PC1, suggesting that PC1 could represent a “quality retention” axis, where higher scores of correlations (*r* = 0.95) indicate better preservation of sensitive nutritional and sensory attributes (Fig. 10). Overall, the PCA confirms that drying parameters significantly affect key quality indicators, and it helps identify trade-offs: for instance, conditions that reduce drying time may compromise flavor and vitamin C if not optimized. The separation of variables along PC1 and PC2 highlights which processing conditions align with desired quality traits, providing a useful framework for optimizing drying protocols.

## 4 Conclusion

This research delves into the significant effects of artificial neural networks to model and forecast the relationship of operational characteristics such as drying time, rehydration ratio, total color change, flavor strength, water activity, vitamin C, and allicin content with the microwave-assisted convective dryer under different drying conditions. The result revealed that the highest drying condition increased the allicin content and vitamin C, while decreasing the total color changes to a minimum at the corresponding time. The drying time increased significantly by 6.25 % at 0.5 m/s and 18.75 % at 1.0 m/s, while maintaining the minimum microwave power and temperature. However, it was observed that increasing the microwave power and temperature decreased flavor content. A significant decrement of 11.90 % in water was observed at 300 W, air velocity of 0.3 m/s, 300 W, and 45 °C. The ANN model offered valuable insights into controlling and understanding the variables influencing the drying process through offering precise forecasts that suited the testing data sets. The rehydration ratio and total color change in the biplot revealed commonalities on the positive sides of PC2. Overall, the ANN application in MW-HA drying demonstrated enhanced drying efficiency in mass transport and quality enhancement at an optimal condition of 45 °C air temperature, 1.0 m/s airflow velocity, and 100 W microwave power. It offered insightful information to help achieve ideal process conditions in the design of cutting-edge drying systems for industrial applications, while offering a new approach for garlic drying technology.

## Limitations of the study

Microwave-convective hot air drying offers numerous advantages compared to conventional drying methods, the drying conditions must be carefully controlled to mitigate potential disadvantages.

## Future scope of the study

Future studies should investigate the impacts of the thermal performance of the drying system on the sample. Furthermore, further research is required to assess the dryers' economic feasibility and optimal performance. Moreover, there is a need to explore techniques that can preserve or improve dried garlic's nutritional, sensory properties and enhance the thermal performance of the dryer.

## CRediT authorship contribution statement

**Hany S. El-Mesery:** Writing – review & editing, Writing – original draft, Validation, Supervision, Methodology, Investigation, Funding acquisition, Formal analysis, Data curation, Conceptualization. **Abdulaziz Nuhu Jibril:** Writing – review & editing, Writing – original draft, Software, Formal analysis, Data curation, Conceptualization. **Ahmed H. ElMesiry:** Visualization, Validation, Software, Methodology, Formal analysis, Data curation. **Zicheng Hu:** Visualization, Validation, Investigation. **Xinai Zhang:** Visualization, Validation. **Amer Ali Mahdi:** Visualization, Validation, Investigation, Conceptualization.

## Declaration of competing interest

The authors declare that they have no known competing financial interests or personal relationships that could have appeared to influence the work reported in this paper.

## Data Availability

Data will be made available on request.

## Appendix A Supplementary data

Supplementary material Image 1
