# Supplementary material for: Artificial neural network and machine learning predictive model for assessing physicochemical properties of garlic slices (Allium sativum L.) during microwave-assisted convective drying process
Source: Food Chem X. 2025 Jun 25;29:102703. doi: 10.1016/j.fochx.2025.102703 (PMC12490423; doi:10.1016/j.fochx.2025.102703)
Supplement: Supplementary file 1 — Supplementary material [file mmc1.docx]

Table S1 – Physicochemical parameters for microwave-assisted convective drying

| P, W | 0.3 m/s |  |  |  | 0.5 m/s |  |  |  | 1.0 m/s |  |  |
| --- | --- | --- | --- | --- | --- | --- | --- | --- | --- | --- | --- |
|  | 45 ^0^C | 55 ^0^C | 65 ^0^C |  | 45 ^0^C | 55 ^0^C | 65 ^0^C |  | 45 ^0^C | 55 ^0^C | 65 ^0^C |
| Rehydration ratio | | | | | | | | | | | |
| 100 | 1.75±0.09a | 1.89±0.09ab | 1.94±0.09bc |  | 1.61±0.09a | 1.75±0.09ab | 1.80±0.09bc |  | 1.48±0.10a | 1.62±0.10ab | 1.67±0.10bc |
| 200 | 1.82±0.09ab | 2.05±0.09cd | 2.09±0.09de |  | 1.68±0.10ab | 1.91±0.10cd | 1.95±0.10de |  | 1.55±0.10ab | 1.78±0.10cd | 1.82±0.10de |
| 300 | 2.00±0.10bc | 2.13±0.10e | 2.17±0.10e |  | 1.86±0.11bc | 1.99±0.11e | 2.03±0.11e |  | 1.73±0.11bc | 1.86±0.11e | 1.90±0.11e |
| Color changes | | | | | | | | | | | |
| 100 | 15.04±0.75a | 18.21±0.75b | 19.72±0.75bc |  | 13.46±0.91a | 16.63±0.91b | 18.14±0.91bc |  | 9.85±0.99a | 13.02±0.99b | 14.53±0.99bc |
| 200 | 18.33±0.92b | 20.75±0.92cd | 23.09±0.92ef |  | 16.75±1.04b | 19.17±1.04cd | 21.51±1.04ef |  | 13.14±1.15b | 15.56±1.15cd | 17.90±1.15ef |
| 300 | 19.76±0.99bc | 21.75±0.99de | 23.79±0.99f |  | 18.18±1.09bc | 20.17±1.09de | 22.21±1.09f |  | 14.57±1.19bc | 16.56±1.19de | 18.60±1.19f |
| Flavor, mg/g dry mass | | | | | | | | | | | |
| 100 | 5.53±0.28a | 5.34±0.28ab | 5.16±0.28ab |  | 5.85±0.27a | 5.66±0.27ab | 5.48±0.27ab |  | 6.29±0.26a | 6.10±0.26ab | 5.92±0.26ab |
| 200 | 5.39±0.27ab | 5.18±0.27ab | 4.99±0.27a |  | 5.71±0.26ab | 5.50±0.26ab | 5.31±0.26a |  | 6.15±0.25ab | 5.94±0.25ab | 5.75±0.25a |
| 300 | 5.22±0.26ab | 5.09±0.26ab | 4.95±0.26a |  | 5.54±0.25ab | 5.41±0.25ab | 5.27±0.25a |  | 5.98±0.25ab | 5.85±0.25ab | 5.71±0.25a |
| Allicin, % | | | | | | | | | | | |
| 100 | 21.26±1.06a | 20.62±1.06cd | 19.58±1.06bc |  | 21.84±1.03d | 21.20±1.03cd | 20.16±1.03bc |  | 22.83±0.98d | 22.19±0.98cd | 21.15±0.98bc |
| 200 | 19.11±0.96bc | 18.54±0.96b | 16.43±0.96a |  | 19.69±0.93bc | 19.12±0.93b | 17.01±0.93a |  | 20.68±0.82bc | 20.11±0.82b | 18.00±0.82a |
| 300 | 16.90±0.85a | 15.69±0.85a | 15.24±0.85a |  | 17.48±0.78a | 16.27±0.78a | 15.82±0.78a |  | 18.47±0.76a | 17.26±0.76a | 16.81±0.76a |
| Vitamin C, mg/g | | | | | | | | | | | |
| 100 | 0.1231±0.006f | 0.1020±0.006e | 0.0870±0.006cd |  | 0.1511±0.005f | 0.1300±0.005e | 0.1150±0.005cd |  | 0.1751±0.004f | 0.1530±0.004e | 0.1390±0.004cd |
| 200 | 0.1020±0.005e | 0.0910±0.005d | 0.0810±0.005bc |  | 0.1300±0.005e | 0.1190±0.005d | 0.1090±0.005bc |  | 0.1540±0.004e | 0.1430±0.004d | 0.1330±0.004bc |
| 300 | 0.0769±0.004ab | 0.0701±0.004a | 0.0787±0.004b |  | 0.1049±0.004ab | 0.0981±0.004a | 0.1067±0.004b |  | 0.1289±0.004ab | 0.1221±0.004a | 0.1307±0.004b |
| Water activity | | | | | | | | | | | |
| 100 | 0.470±0.024b | 0.470±0.024b | 0.460±0.024ab |  | 0.485±0.024b | 0.485±0.024b | 0.475±0.024ab |  | 0.505±0.023b | 0.503±0.023b | 0.495±0.023ab |
| 200 | 0.450±0.023a | 0.450±0.023ab | 0.440±0.023ab |  | 0.465±0.023a | 0.465±0.023ab | 0.455±0.023ab |  | 0.485±0.022a | 0.485±0.022ab | 0.475±0.022ab |
| 300 | 0.420±0.021a | 0.430±0.021ab | 0.420±0.021a |  | 0.435±0.022a | 0.445±0.022ab | 0.435±0.022a |  | 0.455±0.021a | 0.465±0.021ab | 0.455±0.021a |

The values are mean ±SD. The column values with the double letters are statistically similar according to the Duncan Multiple Range Test (DMRT) at p < 0.05

Table S2 – ANN model on statistical data for drying time

| **T, ^0^C** | **P, W** | **V, m/s** | **Dried** | **garlic** |  |  |  |  |
| --- | --- | --- | --- | --- | --- | --- | --- | --- |
|  |  |  | **R^2^** |  |  | **RMSE** |  |  |
|  |  |  | **Testing** | **Validation** | **Training** | **Testing** | **Validation** | **Training** |
| 45 | 100 | 0.3 | 0.9998 | 0.9918 | 0.9990 | 0.0058 | 0.0435 | 0.0113 |
|  | 200 |  | 0.9997 | 0.9988 | 0.9992 | 0.0158 | 0.0239 | 0.0403 |
|  | 300 |  | 0.9999 | 0.9989 | 0.9996 | 0.0130 | 0.0148 | 0.0112 |
| 55 | 100 |  | 0.9998 | 0.9977 | 0.9983 | 0.0161 | 0.0551 | 0.0310 |
|  | 200 |  | 0.9999 | 0.9977 | 0.9986 | 0.0162 | 0.0218 | 0.0216 |
|  | 300 |  | 0.9992 | 0.9971 | 0.9991 | 0.0278 | 0.0256 | 0.0213 |
| 65 | 100 |  | 0.9999 | 0.9992 | 0.9994 | 0.0088 | 0.0152 | 0.0127 |
|  | 200 |  | 0.9991 | 0.9926 | 0.9992 | 0.0436 | 0.0713 | 0.0194 |
|  | 300 |  | 0.9997 | 0.9977 | 0.9994 | 0.0553 | 0.0512 | 0.0040 |
| 45 | 100 | 0.5 | 0.9999 | 0.9981 | 0.9991 | 0.0252 | 0.0422 | 0.0120 |
|  | 200 |  | 0.9998 | 0.9995 | 0.9996 | 0.0274 | 0.0171 | 0.0127 |
|  | 300 |  | 0.9996 | 0.9993 | 0.9995 | 0.0393 | 0.0132 | 0.0320 |
| 55 | 100 |  | 0.9996 | 0.9995 | 0.9996 | 0.0301 | 0.0211 | 0.021 |
|  | 200 |  | 0.9996 | 0.9979 | 0.9998 | 0.0391 | 0.0162 | 0.0312 |
|  | 300 |  | 0.9989 | 0.9991 | 0.9993 | 0.0132 | 0.0152 | 0.0115 |
| 65 | 100 |  | 0.9992 | 0.9947 | 0.9974 | 0.0213 | 0.0289 | 0.0246 |
|  | 200 |  | 0.9997 | 0.9916 | 0.9988 | 0.0184 | 0.0743 | 0.0485 |
|  | 300 |  | 0.9994 | 0.9990 | 0.9967 | 0.0466 | 0.0655 | 0.0237 |
| **45** | **100** | **1.0** | **0.9999** | **0.9993** | **0.9997** | **0.0064** | **0.0136** | **0.0093** |
|  | 200 |  | 0.9997 | 0.9982 | 0.9993 | 0.0277 | 0.0115 | 0.0412 |
|  | 300 |  | 0.9993 | 0.9986 | 0.9997 | 0.0145 | 0.0294 | 0.0421 |
| 55 | 100 |  | 0.9999 | 0.9974 | 0.9989 | 0.0324 | 0.0477 | 0.0128 |
|  | 200 |  | 0.9998 | 0.9986 | 0.9993 | 0.0205 | 0.0270 | 0.0444 |
|  | 300 |  | 0.9997 | 0.9932 | 0.9992 | 0.0152 | 0.0159 | 0.0240 |
| 65 | 100 |  | 0.9996 | 0.9970 | 0.9985 | 0.0636 | 0.0385 | 0.0625 |
|  | 200 |  | 0.9994 | 0.9976 | 0.9985 | 0.0142 | 0.0246 | 0.0171 |
|  | 300 |  | 0.9998 | 0.9909 | 0.9943 | 0.0278 | 0.1212 | 0.0825 |

Table S3 – ANN model on statistical data for rehydration ratio

| **T, ^0^C** | **P, W** | **V, m/s** | **Dried** | **garlic** |  |  |  |  |
| --- | --- | --- | --- | --- | --- | --- | --- | --- |
|  |  |  | **R^2^** |  |  | **RMSE** |  |  |
|  |  |  | **Testing** | **Validation** | **Training** | **Testing** | **Validation** | **Training** |
| 45 | 100 | 0.3 | 0.9996 | 0.9988 | 0.9963 | 0.0015 | 0.0021 | 0.0012 |
|  | 200 |  | 0.9989 | 0.9983 | 0.9934 | 0.0121 | 0.0055 | 0.0146 |
|  | 300 |  | 0.9993 | 0.9942 | 0.9984 | 0.0079 | 0.0053 | 0.0136 |
| 55 | 100 |  | 0.9996 | 0.9995 | 0.9994 | 0.0031 | 0.0023 | 0.0028 |
|  | 200 |  | 0.9996 | 0.9967 | 0.9995 | 0.0014 | 0.0009 | 0.0019 |
|  | 300 |  | 0.9941 | 0.9871 | 0.9962 | 0.0007 | 0.0006 | 0.0038 |
| 65 | 100 |  | 0.9995 | 0.9994 | 0.9998 | 0.0017 | 0.0016 | 0.0020 |
|  | 200 |  | 0.9994 | 0.9991 | 0.9975 | 0.0014 | 0.0013 | 0.0024 |
|  | 300 |  | 0.9993 | 0.9921 | 1.0000 | 0.0004 | 0.0021 | 0.0002 |
| 45 | 100 | **0.5** | 0.9995 | 0.9991 | 0.9999 | 0.0019 | 0.0022 | 0.0014 |
|  | 200 |  | 0.9998 | 0.9982 | 0.9999 | 0.0047 | 0.0029 | 0.0015 |
|  | 300 |  | 0.9997 | 0.9997 | 0.9998 | 0.0028 | 0.0045 | 0.0031 |
| **55** | 100 |  | 0.9999 | 0.9990 | 1.0000 | 0.0034 | 0.0030 | 0.0009 |
|  | **200** |  | **0.9999** | **0.9999** | **1.0000** | **0.0007** | **0.0010** | **0.0005** |
|  | 300 |  | 0.9970 | 0.9960 | 0.9949 | 0.0003 | 0.0003 | 0.0007 |
| 65 | 100 |  | 0.9987 | 0.9982 | 0.9998 | 0.0371 | 0.0155 | 0.0470 |
|  | 200 |  | 0.9998 | 0.9999 | 1.0000 | 0.0005 | 0.0006 | 0.0006 |
|  | 300 |  | 0.9915 | 0.9863 | 0.9904 | 0.0012 | 0.0010 | 0.0011 |
| 45 | 100 | 1.0 | 0.9996 | 0.9993 | 0.9947 | 0.0016 | 0.0018 | 0.0023 |
|  | 200 |  | 0.9997 | 0.9993 | 0.9994 | 0.0042 | 0.0058 | 0.0026 |
|  | 300 |  | 0.9996 | 0.9995 | 0.9993 | 0.0060 | 0.0047 | 0.0070 |
| 55 | 100 |  | 0.9998 | 0.9995 | 0.9991 | 0.0022 | 0.0024 | 0.0023 |
|  | 200 |  | 0.9997 | 0.9995 | 1.0000 | 0.0356 | 0.0105 | 0.0220 |
|  | 300 |  | 0.9969 | 0.9948 | 0.9954 | 0.0009 | 0.0004 | 0.0009 |
| 65 | 100 |  | 0.9997 | 0.9994 | 0.9991 | 0.0026 | 0.0022 | 0.0032 |
|  | 200 |  | 0.9998 | 0.9997 | 0.9998 | 0.0008 | 0.0007 | 0.0009 |
|  | 300 |  | 0.9994 | 0.9994 | 0.9976 | 0.0006 | 0.0004 | 0.0011 |

Table S4 – ANN model on statistical data for color change

| **T, ^0^C** | **P, W** | **V, m/s** | **Dried** | **garlic** |  |  |  |  |
| --- | --- | --- | --- | --- | --- | --- | --- | --- |
|  |  |  | **R^2^** |  |  | **RMSE** |  |  |
|  |  |  | **Testing** | **Validation** | **Training** | **Testing** | **Validation** | **Training** |
| 45 | 100 | **0.3** | 0.9998 | 0.9990 | 1.0000 | 0.0358 | 0.0546 | 0.0462 |
|  | 200 |  | 0.9997 | 0.9996 | 0.9994 | 0.0258 | 0.0119 | 0.0176 |
|  | 300 |  | 0.9938 | 0.9323 | 0.9958 | 0.0495 | 0.1054 | 0.0552 |
| 55 | 100 |  | 0.9996 | 0.9995 | 0.9995 | 0.0621 | 0.0337 | 0.0362 |
|  | 200 |  | 0.9998 | 0.9998 | 0.9997 | 0.0134 | 0.0079 | 0.0181 |
|  | 300 |  | 0.9974 | 0.9966 | 0.9997 | 0.0243 | 0.0161 | 0.0249 |
| **65** | 100 |  | 0.9996 | 0.9996 | 0.9998 | 0.0345 | 0.0303 | 0.0377 |
|  | 200 |  | 0.9997 | 0.9990 | 0.9961 | 0.0324 | 0.0232 | 0.0599 |
|  | **300** |  | **1.0000** | **0.9999** | **1.0000** | **0.0106** | **0.0392** | **0.0118** |
| 45 | 100 | 0.5 | 0.9999 | 0.9997 | 0.9990 | 0.0648 | 0.0494 | 0.0509 |
|  | 200 |  | 1.0000 | 0.9996 | 1.0000 | 0.0201 | 0.0204 | 0.0100 |
|  | 300 |  | 0.9975 | 0.9886 | 0.9976 | 0.0154 | 0.0277 | 0.0214 |
| 55 | 100 |  | 1.0000 | 0.9991 | 1.0000 | 0.0465 | 0.0460 | 0.0156 |
|  | 200 |  | 0.9999 | 0.9997 | 0.9998 | 0.0126 | 0.0140 | 0.0095 |
|  | 300 |  | 0.9964 | 0.9906 | 0.9972 | 0.0189 | 0.0195 | 0.0302 |
| 65 | 100 |  | 0.9992 | 0.9991 | 0.9997 | 0.0479 | 0.0535 | 0.0685 |
|  | 200 |  | 0.9993 | 0.9977 | 1.0000 | 0.0119 | 0.0327 | 0.0062 |
|  | 300 |  | 0.9981 | 0.9932 | 0.9972 | 0.2337 | 0.0947 | 0.2066 |
| 45 | 100 | 1.0 | 0.9998 | 0.9997 | 0.9939 | 0.0396 | 0.0539 | 0.1334 |
|  | 200 |  | 0.9999 | 0.9997 | 0.9959 | 0.0179 | 0.0133 | 0.0507 |
|  | 300 |  | 0.9971 | 0.9861 | 0.9990 | 0.0413 | 0.0546 | 0.0234 |
| 55 | 100 |  | 0.9964 | 0.9919 | 0.9961 | 0.1095 | 0.1137 | 0.0875 |
|  | 200 |  | 0.9995 | 0.9995 | 1.0000 | 0.0356 | 0.0105 | 0.0220 |
|  | 300 |  | 0.9919 | 0.9755 | 0.9923 | 0.1705 | 0.0447 | 0.2036 |
| 65 | 100 |  | 0.9981 | 0.9968 | 0.9913 | 0.2033 | 0.1093 | 0.2286 |
|  | 200 |  | 0.9989 | 0.9947 | 0.9990 | 0.0787 | 0.0393 | 0.0501 |
|  | 300 |  | 0.9995 | 0.9989 | 0.9937 | 0.0697 | 0.0613 | 0.0940 |

Table S5 – ANN model on statistical data for flavor

| **T, ^0^C** | **P, W** | **V, m/s** | **Dried** | **garlic** |  |  |  |  |
| --- | --- | --- | --- | --- | --- | --- | --- | --- |
|  |  |  | **R^2^** |  |  | **RMSE** |  |  |
|  |  |  | **Testing** | **Validation** | **Training** | **Testing** | **Validation** | **Training** |
| 45 | 100 | **0.3** | 0.9996 | 0.9990 | 0.9999 | 0.0030 | 0.0024 | 0.0017 |
|  | 200 |  | 0.9997 | 0.9995 | 0.9992 | 0.0042 | 0.0027 | 0.0041 |
|  | 300 |  | 0.9995 | 0.9994 | 0.9988 | 0.0048 | 0.0033 | 0.0077 |
| 55 | 100 |  | 0.9998 | 0.9995 | 0.9992 | 0.0022 | 0.0023 | 0.0032 |
|  | 200 |  | 0.9996 | 0.9996 | 0.9993 | 0.0020 | 0.0010 | 0.0025 |
|  | 300 |  | 0.9995 | 0.9986 | 0.9996 | 0.0005 | 0.0005 | 0.0002 |
| **65** | 100 |  | 0.9997 | 0.9996 | 0.9999 | 0.0016 | 0.0015 | 0.0018 |
|  | 200 |  | 0.9997 | 0.9992 | 0.9973 | 0.0017 | 0.0014 | 0.0030 |
|  | **300** |  | **1.0000** | **1.0000** | **1.0000** | **0.0005** | **0.0015** | **0.0006** |
| 45 | 100 | 0.5 | 0.9997 | 0.9996 | 0.9984 | 0.0028 | 0.0012 | 0.0025 |
|  | 200 |  | 0.9997 | 0.9984 | 0.9999 | 0.0047 | 0.0032 | 0.0015 |
|  | 300 |  | 0.9998 | 0.9997 | 0.9997 | 0.0015 | 0.0042 | 0.0024 |
| 55 | 100 |  | 0.9997 | 0.9988 | 0.9997 | 0.0041 | 0.0034 | 0.0017 |
|  | 200 |  | 0.9996 | 0.9991 | 0.9998 | 0.0009 | 0.0010 | 0.0007 |
|  | 300 |  | 0.9991 | 0.9984 | 0.9991 | 0.0007 | 0.0004 | 0.0003 |
| 65 | 100 |  | 0.9996 | 0.9992 | 0.9976 | 0.0031 | 0.0020 | 0.0028 |
|  | 200 |  | 0.9994 | 0.9953 | 1.0000 | 0.0006 | 0.0019 | 0.0006 |
|  | 300 |  | 0.9979 | 0.9936 | 0.9939 | 0.0113 | 0.0043 | 0.0109 |
| 45 | 100 | 1.0 | 0.9993 | 0.9982 | 0.9942 | 0.0033 | 0.0052 | 0.0052 |
|  | 200 |  | 0.9934 | 0.9881 | 0.9908 | 0.0091 | 0.0087 | 0.0078 |
|  | 300 |  | 0.9987 | 0.9983 | 0.9999 | 0.0099 | 0.0088 | 0.0078 |
| 55 | 100 |  | 0.9942 | 0.9894 | 0.9974 | 0.0080 | 0.0081 | 0.0093 |
|  | 200 |  | 0.9983 | 0.9974 | 1.0000 | 0.0042 | 0.0023 | 0.0052 |
|  | 300 |  | 0.9966 | 0.9928 | 0.9930 | 0.0025 | 0.0011 | 0.0023 |
| 65 | 100 |  | 0.9966 | 0.9964 | 0.9910 | 0.0109 | 0.0058 | 0.0120 |
|  | 200 |  | 0.9960 | 0.9941 | 0.9926 | 0.0043 | 0.0021 | 0.0022 |
|  | 300 |  | 0.9993 | 0.9988 | 0.9968 | 0.0036 | 0.0028 | 0.0038 |

Table S6 – ANN model on statistical data for Allicin

| **T, ^0^C** | **P, W** | **V, m/s** | **Dried** | **Garlic** |  |  |  |  |
| --- | --- | --- | --- | --- | --- | --- | --- | --- |
|  |  |  | **R^2^** |  |  | **RMSE** |  |  |
|  |  |  | **Testing** | **Validation** | **Training** | **Testing** | **Validation** | **Training** |
| 45 | 100 | 0.3 | 0.9996 | 0.9987 | 0.9996 | 0.0433 | 0.0383 | 0.0181 |
|  | 200 |  | 0.9996 | 0.9994 | 0.9992 | 0.0467 | 0.0369 | 0.0520 |
|  | 300 |  | 0.9997 | 0.9995 | 0.9990 | 0.0560 | 0.0355 | 0.0816 |
| 55 | 100 |  | 0.9996 | 0.9995 | 0.9993 | 0.0438 | 0.0335 | 0.0513 |
|  | 200 |  | 0.9995 | 0.9994 | 0.9987 | 0.0695 | 0.0505 | 0.1195 |
|  | 300 |  | 0.9994 | 0.9993 | 1.0000 | 0.0524 | 0.0403 | 0.0493 |
| 65 | 100 |  | 0.9996 | 0.9996 | 0.9999 | 0.0323 | 0.0293 | 0.0361 |
|  | 200 |  | 0.9999 | 0.9996 | 0.9985 | 0.0298 | 0.0192 | 0.0392 |
|  | 300 |  | 0.9998 | 0.9982 | 1.0000 | 0.0147 | 0.0227 | 0.0082 |
| 45 | 100 | **0.5** | 0.9998 | 0.9985 | 0.9989 | 0.0404 | 0.0430 | 0.0338 |
|  | 200 |  | 0.9998 | 0.9985 | 1.0000 | 0.0583 | 0.0436 | 0.0178 |
|  | 300 |  | 0.9998 | 0.9998 | 0.9997 | 0.0165 | 0.0463 | 0.0262 |
| 55 | 100 |  | 0.9999 | 0.9983 | 0.9995 | 0.0500 | 0.0368 | 0.0211 |
|  | 200 |  | 0.9997 | 0.9997 | 0.9997 | 0.0240 | 0.0621 | 0.0357 |
|  | 300 |  | 0.9996 | 0.9995 | 0.9997 | 0.0667 | 0.0413 | 0.0588 |
| 65 | 100 |  | 0.9986 | 0.9978 | 0.9994 | 0.0659 | 0.0914 | 0.0903 |
|  | **200** |  | **0.9999** | **0.9998** | **0.9999** | **0.0097** | **0.0133** | **0.0075** |
|  | 300 |  | 0.9997 | 0.9916 | 1.0000 | 0.0802 | 0.0353 | 0.0822 |
| 45 | 100 | 1.0 | 0.9995 | 0.9993 | 0.9942 | 0.0480 | 0.0530 | 0.0790 |
|  | 200 |  | 0.9942 | 0.9895 | 0.9911 | 0.1103 | 0.1075 | 0.1011 |
|  | 300 |  | 0.9988 | 0.9986 | 0.9998 | 0.1081 | 0.0930 | 0.0870 |
| 55 | 100 |  | 0.9913 | 0.9864 | 0.9906 | 0.1151 | 0.1126 | 0.1149 |
|  | 200 |  | 0.9930 | 0.9927 | 0.9996 | 0.2759 | 0.1771 | 0.2682 |
|  | 300 |  | 0.9985 | 0.9976 | 0.9964 | 0.4392 | 0.1753 | 0.5955 |
| 65 | 100 |  | 0.9976 | 0.9963 | 0.9903 | 0.2265 | 0.1146 | 0.2501 |
|  | 200 |  | 0.9991 | 0.9989 | 0.9997 | 0.0208 | 0.0274 | 0.0130 |
|  | 300 |  | 0.9988 | 0.9987 | 0.9937 | 0.0294 | 0.0333 | 0.0241 |

Table S7 – ANN model on statistical data for vitamin C

| **T, ^0^C** | **P, W** | **V, m/s** | **Dried** | **garlic** |  |  |  |  |
| --- | --- | --- | --- | --- | --- | --- | --- | --- |
|  |  |  | **R^2^** |  |  | **RMSE** |  |  |
|  |  |  | **Testing** | **Validation** | **Training** | **Testing** | **Validation** | **Training** |
| 45 | 100 | 0.3 | 0.9996 | 0.9990 | 0.9999 | 0.0005 | 0.0004 | 0.0002 |
|  | 200 |  | 0.9995 | 0.9995 | 0.9992 | 0.0006 | 0.0004 | 0.0006 |
|  | 300 |  | 0.9997 | 0.9994 | 0.9989 | 0.0007 | 0.0005 | 0.0011 |
| 55 | 100 |  | 0.9996 | 0.9993 | 0.9995 | 0.0002 | 0.0002 | 0.0003 |
|  | 200 |  | 0.9998 | 0.9993 | 0.9983 | 0.0006 | 0.0004 | 0.0010 |
|  | 300 |  | 0.9993 | 0.9993 | 1.0000 | 0.0005 | 0.0004 | 0.0004 |
| 65 | 100 |  | 0.9996 | 0.9996 | 0.9999 | 0.0006 | 0.0006 | 0.0007 |
|  | 200 |  | 0.9998 | 0.9996 | 0.9985 | 0.0006 | 0.0004 | 0.0007 |
|  | 300 |  | 0.9998 | 0.9990 | 1.0000 | 0.0003 | 0.0004 | 0.0002 |
| 45 | 100 | **0.5** | 0.9997 | 0.9996 | 0.9985 | 0.0004 | 0.0002 | 0.0004 |
|  | 200 |  | 0.9998 | 0.9985 | 0.9999 | 0.0007 | 0.0005 | 0.0002 |
|  | 300 |  | 0.9998 | 0.9997 | 0.9997 | 0.0002 | 0.0006 | 0.0003 |
| 55 | 100 |  | 0.9999 | 0.9981 | 0.9999 | 0.0003 | 0.0002 | 0.0008 |
|  | 200 |  | 0.9997 | 0.9997 | 0.9997 | 0.0002 | 0.0004 | 0.0003 |
|  | 300 |  | 0.9999 | 0.9995 | 0.9997 | 0.0005 | 0.0003 | 0.0005 |
| 65 | 100 |  | 0.9988 | 0.9946 | 0.9955 | 0.0017 | 0.0008 | 0.0022 |
|  | **200** |  | **0.9999** | **0.9998** | **0.9999** | **0.0002** | **0.0003** | **0.0002** |
|  | 300 |  | 0.9996 | 0.9914 | 0.9999 | 0.0001 | 0.0006 | 0.0002 |
| 45 | 100 | 1.0 | 0.9993 | 0.9984 | 0.9942 | 0.0005 | 0.0007 | 0.0008 |
|  | 200 |  | 0.9935 | 0.9883 | 0.9908 | 0.0013 | 0.0013 | 0.0012 |
|  | 300 |  | 0.9987 | 0.9983 | 0.9999 | 0.0014 | 0.0013 | 0.0011 |
| 55 | 100 |  | 0.9978 | 0.9934 | 0.9994 | 0.0004 | 0.0004 | 0.0004 |
|  | 200 |  | 0.9920 | 0.9920 | 0.9993 | 0.0022 | 0.0014 | 0.0021 |
|  | 300 |  | 0.9990 | 0.9979 | 0.9954 | 0.0039 | 0.0015 | 0.0054 |
| 65 | 100 |  | 0.9976 | 0.9963 | 0.9903 | 0.0004 | 0.0002 | 0.0005 |
|  | 200 |  | 0.9994 | 0.9991 | 0.9997 | 0.0004 | 0.0003 | 0.0002 |
|  | 300 |  | 0.9995 | 0.9987 | 0.9931 | 0.0005 | 0.0006 | 0.0004 |

Table S8 – ANN model on statistical data for water

| **T, ^0^C** | **P, W** | **V, m/s** | **Dried** | **garlic** |  |  |  |  |
| --- | --- | --- | --- | --- | --- | --- | --- | --- |
|  |  |  | **R^2^** |  |  | **RMSE** |  |  |
|  |  |  | **Testing** | **Validation** | **Training** | **Testing** | **Validation** | **Training** |
| 45 | 100 | **0.3** | 0.9996 | 0.9989 | 0.9998 | 0.0006 | 0.0004 | 0.0003 |
|  | 200 |  | 0.9996 | 0.9995 | 0.9992 | 0.0006 | 0.0005 | 0.0007 |
|  | 300 |  | 0.9995 | 0.9994 | 0.9987 | 0.0009 | 0.0007 | 0.0017 |
| 55 | 100 |  | 0.9996 | 0.9994 | 0.9992 | 0.0004 | 0.0003 | 0.0005 |
|  | 200 |  | 0.9995 | 0.9995 | 0.9990 | 0.0005 | 0.0004 | 0.0007 |
|  | 300 |  | 0.9995 | 0.9994 | 1.0000 | 0.0003 | 0.0002 | 0.0003 |
| **65** | 100 |  | 0.9995 | 0.9995 | 0.9997 | 0.0002 | 0.0002 | 0.0003 |
|  | 200 |  | 0.9996 | 0.9995 | 0.9981 | 0.0003 | 0.0003 | 0.0004 |
|  | **300** |  | **0.9999** | **0.9992** | **1.0000** | **0.0001** | **0.0006** | **0.0001** |
| 45 | 100 | 0.5 | 0.9996 | 0.9988 | 0.9978 | 0.0004 | 0.0004 | 0.0004 |
|  | 200 |  | 0.9998 | 0.9984 | 0.9997 | 0.0007 | 0.0005 | 0.0003 |
|  | 300 |  | 0.9998 | 0.9997 | 0.9997 | 0.0003 | 0.0009 | 0.0005 |
| 55 | 100 |  | 0.9998 | 0.9985 | 0.9999 | 0.0005 | 0.0004 | 0.0002 |
|  | 200 |  | 0.9998 | 0.9998 | 0.9997 | 0.0001 | 0.0004 | 0.0002 |
|  | 300 |  | 0.9995 | 0.9995 | 0.9997 | 0.0002 | 0.0002 | 0.0003 |
| 65 | 100 |  | 0.9981 | 0.9978 | 0.9926 | 0.0002 | 0.0012 | 0.0030 |
|  | 200 |  | 0.9994 | 0.9994 | 0.9994 | 0.0003 | 0.0004 | 0.0003 |
|  | 300 |  | 0.9952 | 0.9945 | 0.9995 | 0.0018 | 0.0007 | 0.0016 |
| 45 | 100 | 1.0 | 0.9993 | 0.9985 | 0.9942 | 0.0008 | 0.0007 | 0.0008 |
|  | 200 |  | 0.9924 | 0.9862 | 0.9908 | 0.0017 | 0.0016 | 0.0014 |
|  | 300 |  | 0.9986 | 0.9980 | 0.9999 | 0.0020 | 0.0018 | 0.0016 |
| 55 | 100 |  | 0.9902 | 0.9826 | 0.9955 | 0.0013 | 0.0012 | 0.0013 |
|  | 200 |  | 0.9942 | 0.9937 | 0.9998 | 0.0017 | 0.0011 | 0.0018 |
|  | 300 |  | 0.9974 | 0.9967 | 0.9980 | 0.0022 | 0.0009 | 0.0030 |
| 65 | 100 |  | 0.99030 | 0.9905 | 0.9984 | 0.0021 | 0.0012 | 0.0022 |
|  | 200 |  | 0.9984 | 0.9973 | 0.9980 | 0.0013 | 0.0006 | 0.0011 |
